# Spontaneous honeybee behaviour is altered by persistent organic pollutants

**DOI:** 10.1007/s10646-016-1749-0

**Published:** 2016-12-08

**Authors:** Jade Drummond, Sally M. Williamson, Ann E. Fitchett, Geraldine A. Wright, Sarah J. Judge

**Affiliations:** 1 Medical Toxicology Centre, Newcastle University, Newcastle upon Tyne, NE2 4AA UK; 2 Institute of Neuroscience, Newcastle University, Newcastle upon Tyne, NE2 4HH UK

**Keywords:** Honeybee, *Apis mellifera*, Lindane, Pesticide, Polychlorinated biphenyl, Pollutant

## Abstract

The effect of environmental pollutants on honeybee behaviour has focused mainly on currently used pesticides. However, honeybees are also exposed to persistent organic pollutants (POPs). The aim of this laboratory based study was to determine if exposure to sublethal field-relevant concentrations of POPs altered the spontaneous behaviour of foraging-age worker honeybees. Honeybees (*Apis mellifera*) were orally exposed to either a sublethal concentration of the polychlorinated biphenyl (PCB) mixture Aroclor 1254 (100 ng/ml), the organochlorine insecticide lindane (2.91 ng/ml) or vehicle (0.01% DMSO, 0.00015% ethanol in 1M sucrose) for 1–4 days. The frequency of single event behaviours and the time engaged in one of four behavioural states (walking, flying, upside down and stationary) were monitored for 15 min after 1, 2, 3 and 4 days exposure. Exposure to Aroclor 1254 but not lindane increased the frequency and time engaged in honeybee motor activity behaviours in comparison to vehicle. The Aroclor 1254—induced hyperactivity was evident after 1 day of exposure and persisted with repeated daily exposure. In contrast, 1 day of exposure to lindane elicited abdominal spasms and increased the frequency of grooming behaviours in comparison to vehicle exposure. After 4 days of exposure, abdominal spasms and increased grooming behaviours were also evident in honeybees exposed to Aroclor 1254. These data demonstrate that POPs can induce distinct behavioural patterns, indicating different toxicokinetic and toxicodynamic properties. The changes in spontaneous behaviour, particularly the PCB-induced chronic hyperactivity and the associated energy demands, may have implications for colony health.

## Introduction

Exposure to environmental chemicals below the lethal dose threshold can adversely affect honeybee populations (Mullin et al. 2010; Wu et al. 2011). The ability of honeybees to survive or adapt to other environmental influences may be comprised by chemicals which alter the normal behaviour of the honeybee. Understandably the vast majority of studies examining the sublethal effects of environmental chemicals on honeybee behaviour have focussed on currently used pesticides (Desneux et al. 2007). Sublethal doses of pesticides including neonicotinoid, pyrethroid, phenylpyrazole and organophosphate insecticides have been shown to alter honeybee motor activity (Charreton et al. 2015; Williamson et al. 2013b, 2014), learning and memory, (Decourtye et al. 2004; Han et al. 2010b; Lambin et al. 2001; Williamson et al. 2013b), and appetite and foraging behaviour (Colin et al. 2004; Dively et al. 2015; Han et al. 2012, 2010a; Vandame et al. 1995). However, currently used agrochemicals are not the only environmental chemicals in honeybee habitats.

Persistent organic pollutants (POPs) are characterised by their chemical structure and environmental persistence (Jones and de Voogt 1999). They decay very slowly and their lipophilic properties lead to bioaccumulation. As a consequence of their environmental persistence and toxicity in human and wildlife populations, the production of certain POPs is restricted (Lallas 2001). However, these chemicals continue to be detected in the environment (Muir and Howard 2006), including in beeswax collected from honeybee hives (Chauzat and Faucon 2007; Ravoet et al. 2015). The presence of POPs in beeswax is unsurprising given the lipid content of beeswax, the lipophilicity of POPs, and because honeybees bioconcentrate chlorinated compounds. Jan and Cerne (1993) demonstrated that honeybees exposed to polychlorinated biphenyl isomers (PCBs) produced beeswax and honey enriched with these pollutants.

Polluted beeswax can affect the health of honeybee colonies; Wu et al. (2011) and Zhu et al. (2014) reported that beeswax contaminated with pesticide residues affects the health of both larvae and adult honeybees either through direct contact and/or ingesting honey stored in contaminated beeswax cells. There is an assumption that POPs are unlikely to transfer or be present in honey as they are highly lipophilic. However, POPs such as PCBs and organochlorine pesticides are found in honey (Anderson and Wojtas 1986; Herrera et al. 2005; Kujawski et al. 2012; Sanchez-Bayo and Goka 2014; Wang et al. 2010). As they are not routinely monitored, data on PCB concentrations in honey is limited, but nevertheless PCB concentrations of more than 500 ng/g have been detected in honey samples from North America and Europe (Anderson and Wojtas 1986; Herrera et al. 2005). There is much more data on organochlorine pesticides, due to pesticide residue monitoring. Some studies have reported that the organochlorine pesticide lindane (Gamma-HCH), which has been banned for years, is detected more frequently in honey than currently used pesticides (Al-Rifai and Akeel 1997; Blasco et al. 2003; Khan et al. 2004; Kujawski et al. 2012). Blasco et al. (2003) reported that 95% of the samples tested from Portugal contained lindane at concentrations of up to 4.3 ng/g (mean 1.3 ng/g). Similar lindane concentrations have been detected in honey samples from Spain (maximum concentration 6 ng/g) (Herrera et al. 2005) and Poland (3.9–4.7 ng/g) (Kujawski et al. 2012). Furthermore, in a recent study which examined data from multiple countries, Sanchez-Bayo and Goka (2014) reported that of the forty pesticides assessed, lindane had the highest residue load in honey. Sanchez-Bayo and Goka (2014) combined the residue load data, prevalence and toxicity (LD50) of the pesticides and calculated that the highest dietary risks to honeybees were for the neonicotinoid thiamethoxam and lindane. Therefore honeybees feeding on honey are at risk of exposure to lindane and other chlorinated pollutants.

The aim of this study was determine if exposure to Aroclor 1254, a PCB isomers mixture, and the organochlorine pesticide lindane altered spontaneous behaviour in winter honeybees. Aroclor 1254, which has a relatively high chlorine content, contains PCB isomers including those which have been detected in honey (Herrera et al. 2005) and in honeybees (Anderson and Wojtas 1986). Honeybees were provided with sucrose solution containing sublethal concentrations of the POPs, which were comparable to the concentrations detected in honey and therefore similar to the concentrations honeybees are potentially consuming in the field. We identified how exposure to these two substances influenced motor activity in honeybees using a protocol modified from an original study of motor function in honeybees by Maze et al. (2006). This initial study paved the way for several subsequent studies that have successfully used this technique to characterise the influence of chemicals on honeybee activity (Mustard et al. 2010; Williamson et al. 2013b).

## Materials and methods

### Insects

European worker honeybees (*Apis mellifera* var. Buckfast) were maintained in an indoor colony with access to the outdoors. Between February and March 2013, adult workers were collected from the hive as they flew out. Honeybees were collected in small plastic pots, briefly anaesthetised by chilling on ice and then transferred into 16.5 cm × 11 cm × 6.5 cm plastic boxes bottom-lined with tissue paper (Williamson et al. 2014; Williamson and Wright 2013). Three 2 ml microcentrifuge tubes with four evenly spaced 2 mm holes were filled with 1 M sucrose solution containing chemicals (see below) and pushed through holes in the sides of the boxes. Honeybees were placed into the boxes to feed ad libitum on the treatment solutions, which were replenished daily. Boxes were kept on the laboratory bench at room temperature and exposed to a standard UK daylight cycle (~ 9 h daylight).

### Chemicals

Aroclor 1254 (Sigma-Aldrich), which is highly viscous, was diluted 1 : 3 with ethanol and then this was diluted further with dimethyl sulfoxide (DMSO) to make a 1 mg/ml stock solution. On the day of administration the stock was diluted to 100 ng/ml 1M sucrose (307 nM Aroclor 1254, 0.01% DMSO, 0.00015% ethanol). Lindane (Sigma-Aldrich) was diluted with DMSO to produce a 0.291 mg/ml stock solution and this was diluted on the day of administration to 2.91 ng/ml 1M sucrose (10 nM, 0.001% DMSO). A vehicle solution to match the DMSO and ethanol concentrations in the Aroclor 1254 solution (0.01% DMSO, 0.00015% ethanol in 1M sucrose) was prepared on the day of administration. As the DMSO concentration in the vehicle solution was greater than the DMSO concentration in the lindane solution, an additional vehicle solution was not used. Previous experiments in this laboratory have demonstrated that DMSO does not affect behaviour at concentrations of 0.1% or less (Williamson et al. 2013a).

### Preliminary tests

Two pilot studies were conducted to confirm that concentrations of chemicals to be used in the behavioural tests did not significantly affect mortality and to determine consumption. First, honeybees were exposed to 100 ng/ml Aroclor 1254 or vehicle for 4 days (*n* = 20–21 honeybees per box, one box per treatment per cohort, 2 cohorts) and mortality and consumption were recorded daily. The concentration of 100 ng/ml was selected because this value had been reported in pollen (Morse et al. 1987). Furthermore, the concentrations of individual PCB isomers in 100 mg/ml Aroclor 1254 ranged from 0.01–13.59 ng/ml (calculated using the percentage weights (Agency for Toxic Substances and Disease Registry 2000)), and were therefore comparable to individual PCB isomer values in honey (mean 0.28–25.4 ng/g) (Herrera et al. 2005). A second group of honeybees were exposed to 0.29, 2.91 or 29.1 ng/ml lindane for 4 days and mortality was recorded (*n* = 14–16 honeybees per box, one box per treatment, 1 cohort). The upper lindane concentration, which equates to 100 nM, has been used in previous pilot studies to determine sublethal pesticide concentrations (Williamson et al. 2014). The lindane concentration of 2.91 ng/ml, was comparable to the concentrations reported in honey samples (0–6 ng/g) (Blasco et al. 2003; Herrera et al. 2005; Kujawski et al. 2012).

For behavioural studies, honeybees (*n* = 192) were exposed to vehicle, Aroclor 1254 (100 ng/g 1M sucrose) or lindane (2.91 ng/g 1M sucrose) for 1–4 days (*n* = 15 honeybees per box, two boxes per treatment per cohort, 4 cohorts). Thus, the concentrations of chemicals offered are similar to POP concentrations honeybees potentially come into contact with in the field.

### Effects on honeybee behaviours

Behavioural observations were recorded using a method adapted from Maze et al. (2006) and performed by Williamson et al. (2013b). Individual honeybees (*n* = 192) were placed in small plastic pots and briefly chilled on ice before transferring each honeybee to a petri dish. Following a 10 min acclimatisation period, the honeybee was observed continuously for 15 min. Behavioural observations were recorded using Noldus Observer software (Noldus Information Technology, Version 5.0). Seven single event behaviours were quantified (Table 1). Single event behaviours were discrete events and mutually exclusive. For example, if a honeybee lifted and extended its leg 5 times, this was counted as 5 leg extension bouts. The duration of the time spent in one of four mutually exclusive states was also recorded (Table 1). Time spent in a behavioural state was converted to a percentage of the observation period by the Observer software. Honeybees could be recorded as stationary (standing still) or walking but at the same time exhibit a behavioural event such as wing fanning or abdominal spasms. Sixteen honeybees in total were observed per treatment group per time point (1,2,3 and 4 days of exposure). Once observed, honeybees were not reused.

**Table 1:** Definitions of spontaneous honeybee behaviours observed in petri dish over 15 min

Behaviour	Description
**Events**	
Flying	In flight in arena
Walking	Walking and not displaying any other behaviour
Upside down	On ventral surface and attempting to perform righting reflex
Abdominal spasm	Abdomen contracting and relaxing
Leg extension	Standing still and lifting and extending leg/legs
Grooming	Rubbing antennae, body or proboscis with legs
Still	Standing still and not displaying any other behaviour
**States**	
Flying	In flight in arena
Walking	Walking but may display another behaviour
Upside down	On ventral surface and attempting to perform righting reflex
Stationary	Standing still but may display another behaviour

### Statistical analysis

**Fig. 1:**
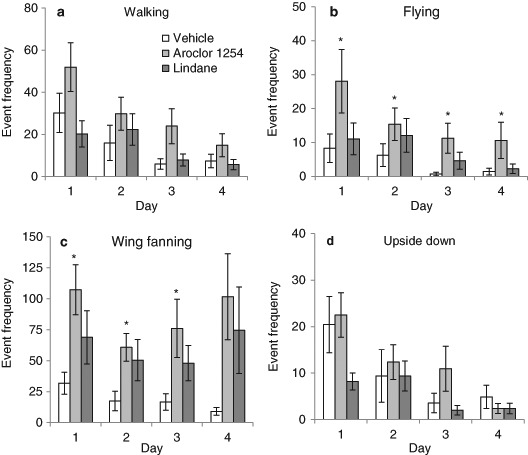
Honeybee exposure to Aroclor 1254, but not lindane, increased the frequency of Factor 1 behaviours (motor-activity). Aroclor 1254 (100 ng/ml, *n* = 64) increased the frequency of walking (**a**), flying (**b**) wing fanning (**c**) and upside down behaviours (**d**) in comparison to vehicle exposure (*n* = 64), whereas exposure to lindane (2.91 ng/ml, *n* = 64) did not. The frequency of Factor 1 behaviours decreased with each day but this decline in activity was observed in all chemical groups. Note inter-panel differences in y-axis scales. Mean ± SEM, * significantly different from vehicle group, *P* < 0.025, Mann-Whitney test

**Fig. 2:**
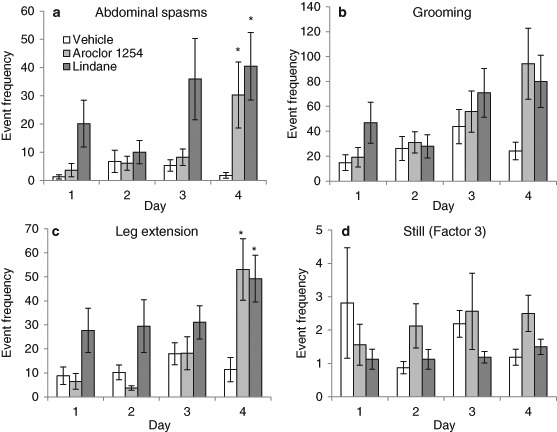
Honeybee exposure to persistent organic pollutants affected the frequency of Factor 2 (**a**–**c**) and Factor 3 behaviours (**d**). From Day 1, honeybees exposed to lindane (2.91 ng/ml, *n* = 64) had more frequent bouts of abdominal spasms (**a**), grooming (**b**) and leg extensions (**c**) than honeybees exposed to vehicle (*n* = 64). Honeybees exposed to Aroclor 1254 (100 ng/ml, *n* = 64) did not exhibit an increase in abdominal spasms (**a**), grooming (**b**) and leg extensions (**c**) until Day 4 of exposure. Being still and not displaying any other behaviour (Factor 3, d) was not affected by Aroclor 1254 exposure but decreased with lindane exposure. Note inter-panel differences in y-axis scales. Mean ± SEM, * significantly different from vehicle group, *P* < 0.025, Mann-Whitney test

**Fig. 3:**
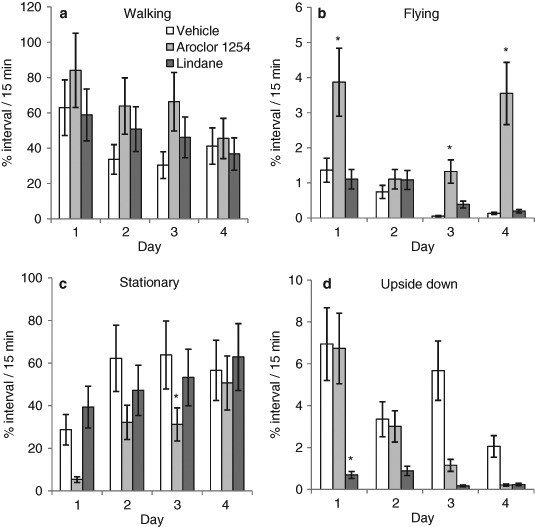
Exposure to Aroclor 1254 (100 ng/ml, *n* = 64), but not lindane (2.91 ng/ml, *n* = 64) altered the percentage of the observation period honeybees spent in one of four behavioural states. Exposure to Aroclor 1254 increased the time spent walking (**a**) and (**b**) flying and decreased the time being stationary (**c**) in comparison to vehicle exposure (*n* = 64). Time spent being upside down was not statistically affected by treatment (**d**). Time spent walking (**a**), flying (**b**) and being upside down (**d**) decreased with each day of exposure whereas, time spent being stationary increased with each day of exposure. Note inter-panel differences in y-axis scales. Mean ± SEM, *significantly different from vehicle group, *P* < 0.025, Mann-Whitney test

As the behavioural events recorded during the observation sessions were mutually exclusive and therefore correlated, the dimensionality of the frequency data was reduced. Factor analysis was performed on the frequency data using the principal components method of factor extraction with a Varimax rotation (Hurst et al. 2014). The generated factor scores were analysed using a multivariate analysis of variance (MANOVA) with Factor as the variable and chemical and day of exposure as fixed factors, with post hoc comparisons. For each day, pairwise comparisons against the vehicle exposed group were made on the frequency data of single event behaviours using a non-parametric Mann-Whitney U test with a adjustment for multiple comparisons. Percentage of interval data were analysed using a non-parametric Kruskal-Wallis test with chemical or day as the grouping variable. If test results were significant pairwise comparisons against the vehicle exposed group were made using a Mann-Whitney U test, with a Bonferonni adjustment for multiple comparisons. Results in Figs. 1–3 are displayed as mean values with standard error of the mean bars.

## Results

### Preliminary tests

Honeybee mortality rate was not affected by repeated daily exposure to Aroclor 1254 (100 ng/ml) (Log rank, *χ* ^2^ (1) = 0.02, NS, Online Resource 1) but was affected by repeated exposure to lindane (0.29, 2.91 or 29.1 ng/ml; Log rank, *χ* ^2^ (2) = 0.97, *P* < 0.01, Online Resource 1). Only 29.1 ng/ml lindane affected mortality (multiple comparison post hoc tests, *P* = 0.026), and so 2.9 ng/ml was considered sublethal. Solution consumption per day (weight of solution consumed by box of honeybees/number of living honeybees) did not change significantly over the course of the 4 days (Repeated Measures General Linear Model, day main effect, NS, Online Resource 1). However, in comparison to vehicle consumption (0.2 g/honeybee/day), Aroclor 1254 consumption was significantly lower (0.13 g/honeybee/day; treatment main effect, *F*_1,2_ = 372, *P* < 0.01; Online Resource 1).

### Spontaneous honeybee behaviours

Factor analysis revealed that Factor 1 was positively correlated with the frequency of walking, flying, wing fanning and upside down bouts and negatively correlated with grooming and leg extensions (Table 2). Factor 2 was positively correlated with the frequency of bouts of grooming, leg extensions and abdominal spasms (Table 2). Factor 3 was correlated with the frequency of being still but was not correlated with other behaviours.

**Table 2:** Factor analysis on the frequency of bouts of spontaneous honeybee behaviours observed in petri dish over 15 min

	Factor		
	1	2	3
% Variance	42.4	21.6	13.4
Walking	**.964**	−.188	.089
Flying	**.897**	−.113	−.092
Upside down	**.804**	−.232	.221
Wing fanning	**.619**	.011	−.489
Abdominal spasms	−.074	**.924**	−.009
Grooming	−.105	**.908**	.038
Leg extension	−.264	**.633**	.226
Still	.122	.150	**.878**

Fit accomplished using a Varimax rotation (5 iterations). Variables with strong contributions to each factor are in bold

### Effect of POPs on the frequency of motor-activity behaviours

Honeybees exposed to vehicle exhibited similar numbers of bouts of walking, wing fanning and upside down behaviours during the observation period (Factor 1 behaviours, Figs. 1a, c, d). Flying was exhibited less frequently (Fig. 1b). Chemical exposure significantly affected the number of bouts of Factor 1 behaviours. Aroclor 1254 exposure increased the frequency of these motor-activity behaviours compared to vehicle exposure (Figs. 1a–d; Table 3). On Day 1 the frequency of flying and fanning bouts was 3 times greater in honeybees exposed to Aroclor 1254 than those exposed to vehicle. By day 4 the average frequency of flying and fanning bouts was over 7 times greater in honeybees exposed to Aroclor 1254 than those exposed to vehicle. In contrast, lindane exposure did not affect the frequency of honeybee motor-activity behaviours (Figs. 1a–d; Table 3).

**Table 3:** Outcomes from statistical analysis of the frequency of spontaneous behaviours observed in honeybees orally exposed to Aroclor 1254, lindane or vehicle (Chemical) for 1,2,3 or 4 days (Day)

MANOVA model term	*F (df)*	*P* value	Bonferonni comparisons with vehicle, *P* value		
			Aroclor 1254	lindane	
**Chemical**					
Factor 1	9.6 (2180)	**.000**	**.000**	.796	
Factor 2	13.6 (2180)	**.000**	**.004**	**.000**	
Factor 3	3.4 (2, 180)	**.035**	.633	**.029**	
			**Bonferonni comparisons with Day 1**, ***P value***		
			**Day 2**	**Day 3**	**Day 4**
**Day**					
Factor 1	5.2 (3180)	**.002**	.169	**.006**	**.004**
Factor 2	6.4 (3180)	**.000**	1.00	.503	**.003**
Factor 3	0.4 (3180)	.769	1.00	1.00	1.00
Chemical * Day					
Factor 1	0.5 (6180)	.812			
Factor 2	2.2 (6180)	**.042**			
Factor 3	0.9 (6180)	.535			

Factor scores generated from Factor Analysis (Table 2) were analysed using a multivariate analysis of variance (MANOVA) followed by Bonferonni post hoc comparisons. Significant values are in bold

Factor 1 behaviours were affected by repeated daily exposure (Table 3, MANOVA effect of day); the number of bouts of walking, flying, wing fanning and upside down behaviours decreased with each experimental day (Figs. 1a–d). However, the decline in Factor 1 behaviours with each day was independent of chemical exposure (Table 3, MANOVA Chemical * Day interaction).

### Effect of POPs on the frequency of other behaviours

Honeybees exposed to vehicle exhibited similar numbers of bouts of grooming and leg extensions but rarely displayed abdominal spasms (Factor 2 behaviours, Figs. 2a–c). Aroclor 1254 exposure and lindane exposure increased the frequency of grooming, leg extensions and abdominal spasms (Factor 2 behaviours, Figs. 2a–c; Table 3). Factor 2 behaviours were affected by repeated daily exposure and this was dependent on chemical exposure. On Day 1 abdominal spasms were evident in honeybees exposed to lindane (Fig. 2a) and the average frequency of bouts of grooming and leg extensions was 3 times greater in honeybees exposed to lindane than those exposed to vehicle or Aroclor 1254 (Figs. 2b–c). By Day 4, abdominal spasms were also observed in honeybees exposed to Aroclor 1254 and the average frequency of bouts of grooming and leg extensions was 4 times greater in honeybees exposed to lindane and to Aroclor 1254 than those exposed to vehicle (Figs. 2b–c).

Honeybees exposed to vehicle rarely displayed bouts of being still and not displaying any other behaviour (Factor 3 behaviours; Fig. 2d). Chemical exposure affected the frequency of being still (Fig. 2d; Table 3). Lindane exposure decreased slightly the frequency of being still and not displaying any other behaviour, Aroclor 1254 did not (Fig. 3d, Table 3). The frequency of bouts of being still was not affected by day of exposure (Fig. 2d; Table 3).

### Effect of POPs on time spent engaged in behaviours

Honeybees exposed to vehicle (*n* = 64) spent on average 42.1 ± 5.3% of the observation period walking and 52.8 ± 6.6% in a stationary state (with or without another behaviour, Fig. 3). Only 0.6 ± 0.1% of the time was spent flying and 4.5 ± 0.6% of the time was spent in an upside down state (Figs. 3b, d). Chemical exposure significantly affected the time engaged in all four behavioural states with Aroclor 1254 exposure, but not lindane, increasing the time spent walking and flying and decreasing the time being stationary (Fig. 3; Table 4). Regardless of chemical treatment, the time spent walking, flying and being upside down decreased and time spent being stationary increased with each experimental day (Fig. 3; Table 4).

**Table 4:** Outcomes from statistical analysis of the percentage of the observation period honeybees spent in one of four behavioural states

	Kruskal-Wallis Test		Mann-Whitney tests			
			Vehicle vs. Aroclor 1254		Vehicle vs. lindane	
	*χ* ^2^ (*df*)	*P*	U	*P*	U	*P*
**Chemical**						
Walking	9.5 (2)	**.009**	1432	**.003**	1888	.441
Flying	25.6 (2)	**.000**	1108	**.000**	1804	.166
Upside down	6.4 (2)	**.041**	1821	.269	1829	.276
Stationary	10.2 (2)	**.006**	1488	**.007**	2015	.873

			Day 1 vs. Day2		Day 1 vs. Day 3		Day 1 vs. Day 4	
			U	*P*	U	*P*	U	*P*
**Day**								
Walking	8.6 (3)	**.035**	885	.050	925	.095	721	**.002**
Flying	12.8 (3)	**.005**	995	.231	822	**.010**	746	**.001**
Upside down	22.3 (3)	**.000**	817	**.013**	621	**.000**	568	**.000**
Stationary	14.3 (3)	**.003**	838	**.017**	742	**.002**	665	**.000**

Honeybees were orally exposed to Aroclor 1254, lindane or vehicle (Chemical) for 1,2,3 or 4 days (Day). Percentage of interval was analysed using a non-parametric Kruskal-Wallis test with chemical or day as the grouping variable followed by post hoc Mann-Whitney U tests. Significant values are in bold

## Discussion

In this study, honeybees were maintained in plastic boxes and administered chemicals through their only available food source: sucrose solution. This method is less time-consuming and stressful than anaesthetising, restraining and feeding honeybees which has been employed in some other studies (Williamson et al. 2013a, b) and is arguably more relevant to field exposure as honeybees are voluntarily consuming the chemical (Jan and Cerne 1993). However, there are a number of limitations to this method. Firstly, individual chemical consumption can only be estimated. We estimated that honeybees ingested on average 132 µl of 0.1 ng/µl (0.1 ppm) Aroclor 1254 per day or 13 ng/day (Aroclor 1254 consumed by box/number of living honeybees in box) but individual ingestion would have differed between honeybees. Secondly, to ensure that honeybees were exposed to the test chemicals, they were not offered an alternative food source. Given a choice, honeybees in the field may not consume food contaminated with POPs and opt for an uncontaminated food source. The prevalence of POPs in the environment, however, means uncontaminated food may not be available. Thirdly, the maintenance of bees in experimental boxes may affect their feeding behaviour and differ from honeybees in the field. If feeding was increased this could potentially mean honeybees in this study were exposed to higher levels of POPs than those in the field. This may be negated to some extent as the POP concentrations used in this study (Aroclor 1,254,100 ng/ml; lindane 2.91 ng/ml) are lower than those found in some honey samples (PCBs 500 ng/g; lindane 4 ng/g (Blasco et al. 2003; Herrera et al. 2005)). Nevertheless there is a need for further studies to determine POP exposure in honeybees in the field.

Honeybees exhibited various spontaneous behaviours during daily observation periods. Motor-activity behaviours of walking, flying, wing-fanning and upside down behaviours were positively correlated with each other. Grooming, leg extensions and abdominal spasms were also positively correlated. One day oral exposure to field-relevant sublethal concentrations of the PCB mixture Aroclor 1254 increased the frequency of and time spent engaged in honeybee motor-activity behaviours. The Aroclor 1254-induced hyperactivity persisted with repeated exposure in the four subsequent days. Repeated exposure to Aroclor 1254 also increased abdominal spasms and grooming and leg extension behaviours in the honeybees. One day oral exposure to field-relevant sublethal concentrations of the organochlorine pesticide lindane did not significantly affect honeybee motor-activity behaviours but did cause abdominal spasms and increased the frequency of grooming and leg extension behaviours.

The motor-activity behaviours exhibited by the honeybees (walking, flying, wing-fanning) and the positive correlation between the motor-activity behaviours is consistent with previous reports (Hurst et al. 2014; Williamson et al. 2014). Likewise, the observation that the honeybees spent much of the period walking and exhibited frequent bouts of walking, and that they also exhibited frequent bouts of flying but actually spent a very small percentage of their time flying, has also been reported previously (Hurst et al. 2014; Williamson et al. 2014). Motor-activity behaviours declined over the course of the 4 day experimental period, regardless of chemical exposure group.

Exposure to the PCB mixture Aroclor 1254 increased both the frequency of and time engaged in motor-activity behaviours. This was evident after 1 day of exposure and persisted over the course of the experimental period. Significant hyperactivity and increased general arousal has been reported in *Drosophila* administered methamphetamine, a dopamine transporter inhibitor, and in *fmn*, a *Drosophila* dopamine transporter mutant (Andretic et al. 2005; Kume et al. 2005). Given that PCBs, including those present in Aroclor 1254, inhibit the mammalian dopamine transporter (Wigestrand et al. 2013), it is possible that Aroclor 1254 could also block dopamine reuptake in the honeybee. An increase in dopaminergic transmission is consistent with hyperactivity in vertebrates and in invertebrates (Andretic et al. 2005; Puhl and Mesce 2008; Sawin et al. 2000; Zhuang et al. 2001). Indeed, it has been suggested that alterations in the dopamine transporter may contribute to the PCB-induced hyperactivity observed in rodents and humans (Lee et al. 2012; Verner et al. 2015). It should be noted however, that PCBs have multiple targets in the mammalian nervous system (Inglefield et al. 2001; Inglefield and Shafer 2000; Mariussen and Fonnum 2001) and this could also be true in invertebrates.

Exposure to lindane (2.91 ng/ml, 10 nM) did not affect the frequency and duration of motor-activity behaviours. Wing fanning frequency did appear to increase with lindane but this was not statistically significant. We had predicted that lindane would increase motor-activity as it well-established from in vitro studies that lindane blocks insect inhibitory GABA-gated and glutamate-gated chloride channels and thereby reduces neuronal inhibition (Ihara et al. 2005; Lees and Calder 1996). The lack of effect on motor activity may be because we examined a low field-relevant concentration of lindane, and higher subtoxic concentrations may well induce hyperactivity, reported in mammals (Llorens et al. 1989). However, the field-relevant concentration was sufficient to induce other behavioural effects (see below). It is also possible that despite evidence that direct application of lindane to nervous tissue blocks inhibitory receptors (Ihara et al. 2005; Lees and Calder 1996), toxicokinetic factors which are relevant when the whole insect is exposed, may prevent lindane from significantly affecting the neural regulation of motor activity.

Abdominal spasms were rarely observed in honeybees exposed to vehicle indicating that they are associated with chemical exposure and are a symptom of acute toxicity (Hurst et al. 2014). Similar abdominal spasms/abdominal dragging have been described previously in honeybees following acute exposure to pesticides and toxins (Hurst et al. 2014; Williamson et al. 2014). Abdominal behaviours could be interpreted as the gut’s response to detecting a potentially harmful xenobiotic. Certainly in mammals, if something ingested is detected as harmful the body responds with gastrointestinal muscle contractions to expel it (Furness et al. 1999). The abdominal spasms exhibited by honeybees exposed to lindane and Aroclor 1254 strongly indicate that these compounds are toxic to honeybees and cause them to present symptoms of ‘malaise’ behaviour.

Abdominal spasms were positively correlated with grooming and leg extension behaviours as observed by Hurst et al. (2014). The peculiar leg extension behaviours we observed in this study were like grooming, but different from grooming of the abdomen with the back legs as defined in previous work (Hurst et al. 2014). This could indicate that POPs also have specific effects on targets in the nervous system governing grooming. Alterations in time spent grooming is often strongly associated with chemical exposure in honeybees; indeed it is often the only aspect of spontaneous behaviour that is affected (Hurst et al. 2014; Oliver et al. 2015; Williamson et al. 2014). Changes in grooming behaviour in this assay with honeybees are often a function of the concentration of a drug or chemical; for example, in the original study of Maze et al. (2006), honeybees fed with 10–25% ethanol in sucrose exhibited more grooming. The fact that honeybees spend more time grooming when they have ingested chemicals could be a reaction to the discomfort caused by toxicosis (Hurst et al. 2014) or a function of the specific action of ingested chemicals on targets within the nervous system such as GABA receptors (Maze et al. 2006).

## Conclusion

Despite the prevalence of POPs in the environment and their presence in honey and beeswax, this is the first time their effects on honeybee behaviour have been described. Just one day exposure to a field-relevant concentration of lindane was long enough to induce malaise-like symptoms in honeybees. Perhaps of more concern is that exposure to a field-relevant concentration of the PCB mixture Aroclor 1254, did not appear to be immediately detected but was capable of inducing significant hyperactivity. After 4 days of Aroclor 1254 exposure the frequency of flying and wing fanning increased by seven times. Such high-level energy demands may have significant implications on the health of the individual honeybee and, therefore, the colony.

## Electronic supplementary material

Online Resource
