# Supplementary material for: Spontaneous honeybee behaviour is altered by persistent organic pollutants
Source: Ecotoxicology. 2016 Dec 8;26(1):141–50. doi: 10.1007/s10646-016-1749-0 (PMC5241328; doi:10.1007/s10646-016-1749-0)
Supplement: Supplementary file 1 — Online Resource [file 10646_2016_1749_MOESM1_ESM.docx]

**Spontaneous honeybee behaviour is altered by persistent organic pollutants**

**Ecotoxicology**

Jade Drummond*†, Sally M. Williamson†, Ann E. Fitchett*†, Geraldine A. Wright† and Sarah J. Judge*†1

*Medical Toxicology Centre, Newcastle University, Newcastle upon Tyne, NE2 4AA, UK

†Institute of Neuroscience, Newcastle University, Newcastle upon Tyne, NE2 4HH, UK

[s.j.judge@ncl.ac.uk](mailto:s.j.judge@ncl.ac.uk)

**Online Resource 1: Effect of exposure to persistent organic pollutants on honeybee survival**

Kaplan-Meier survival curves showing that (a) oral exposure to Aroclor 1254 (100 ng / ml in 1M sucrose) did not affect the honeybee mortality rate in comparison to vehicle (0.01% DMSO, 0.00015% ethanol in 1M sucrose; Log rank, χ^2^ _(1)_ = 0.02, *NS*) and (b) exposure to lindane did affect honeybee mortality rate (Log rank, χ^2^ _(2)_ = 0.97, *P* < 0.01) but only at 29.1 ng / ml (multiple comparisons, *P* = 0.026). (c) Consumption of Aroclor 1254 (closed circles) and vehicle (open circles) did not change over time (Repeated Measures General Linear Model, day main effect, NS) but Aroclor 1254 consumption was less than vehicle consumption (treatment main effect, F1,2 = 372, P < 0.01). Each line represents one treatment box (11–21 honeybees).
